# CexE Is a Coat Protein and Virulence Factor of Diarrheagenic Pathogens

**DOI:** 10.3389/fmicb.2020.01374

**Published:** 2020-06-30

**Authors:** Zachary P. Rivas, Kacey M. Talbot, Leidy C. Merselis, Ryan M. McCormack, Becky Adkins, George P. Munson

**Affiliations:** Department of Microbiology and Immunology, University of Miami Miller School of Medicine, Miami, FL, United States

**Keywords:** ETEC, *C. rodentium*, pathogenesis, virulence factors, CexE

## Abstract

CexE is a 12 kDa protein that was originally reported to be present in just three strains of enterotoxigenic *Escherichia coli* (ETEC); a frequent cause of diarrheal illnesses worldwide. However, an examination of sequenced genomes has revealed that CexE is actually present in a majority of ETEC strains. In addition, homologs of CexE are present in enteroaggregative *E. coli* (EAEC), *Yersinia enterocolitica*, *Providencia alcalifaciens*, and *Citrobacter rodentium*. Although it has been hypothesized that CexE and its homologs are virulence factors, this has yet to be tested. Thus the primary aim of this study was to determine if these proteins contribute to pathogenicity. Our secondary aim was determine if they are secreted coat proteins. Here we report that all neonatal mice infected with a wild-type strain of *C. rodentium* perished. In contrast a *cexE* mutant was significantly attenuated with 45% neonate survival. In adult mice the wild-type strain reached significantly higher loads in the large intestines and were shed in greater numbers than *cexE* mutants. Secretion of the CexE homolog in EAEC is dependent upon an atypical Type I secretion system that accepts its client from the periplasm rather than the cytoplasm. Insertion mutants of *cexC*, the putative ATPase of the CexE secretion system, were attenuated in our murine model. *In vitro* we found that CexC is required for the secretion of CexE to the outer membranes of both ETEC and *C. rodentium*. Secretion is not constitutive because CexE accumulates in the periplasm when the two pathogens are cultured under noninducing conditions. Although secretion conditions differ between ETEC and *C. rodentium*, secreted CexE remains predominantly associated with the outer membranes of both species. In aggregate these findings demonstrate that CexE is a secreted coat protein and virulence factor that promotes colonization of host intestinal tissues by enteric pathogens.

## Introduction

Approximately half a million infants and children perish from diarrheal diseases every year (World Health Organization, 2017). For citizens of low–income countries diarrheal diseases are projected to remain among the ten leading causes of death through 2030 (Mathers and Loncar, 2006). Of the many viral and bacterial pathogens that cause diarrhea enterotoxigenic *Escherichia coli* (ETEC) is one of the most frequent causes with at least 280 million people sickened annually; most in low-income nations (Qadri et al., 2005). ETEC causes diarrhea by elaborating enterotoxins that disrupt the normal flow of ions across the apical membranes of enterocytes. This disruption leads to a net loss of water into the lumen of the intestinal tract resulting in profuse watery diarrhea (Fleckenstein et al., 2010). In some instances ETEC induced diarrhea results in severe dehydration that can result in death if not adequately addressed.

Enterotoxins are the penultimate cause of diarrhea but ETEC must also colonize the gastrointestinal tract to cause disease. Colonization is itself dependent upon the expression of pili that function as adherence factors facilitating the direct attachment of ETEC to enterocytes (Evans et al., 1978; Kernéis et al., 1992). Although ETEC are heterogeneous and there are numerous antigenically distinct pilus types, many are positively regulated at the level of transcription by the ETEC virulence regulator Rns (CfaD) (Caron et al., 1989; Caron and Scott, 1990; Bodero et al., 2008; Bodero and Munson, 2016). *In vitro* DNase I footprinting studies have shown that each Rns activated pilin promoter contains a Rns DNA binding site immediately upstream of the promoter’s -35 hexamer (Munson and Scott, 1999; Bodero et al., 2008; Bodero and Munson, 2016). In most cases the promoter proximal binding site is usually accompanied by one or more additional Rns binding sites further upstream. However site directed mutagenesis studies have shown that occupancy of the promoter proximal site has the greatest contribution to promoter activation (Munson and Scott, 1999; Bodero and Munson, 2016).

An *in silico* analysis of the genome of ETEC strain H10407 -a strain that has been used in several human challenge studies- led to the identification of two Rns binding sites upstream of *cexE* (Dupont et al., 1971; Evans et al., 1978; Satterwhite et al., 1978; Pilonieta et al., 2007; Crossman et al., 2010). As with pilin promoters, one of the binding sites was located immediately upstream of the -35 hexamer of *cexEp* and the other further upstream. As expected from the positions of its binding sites, Rns was found to activate the expression of *cexE*. Although *cexE* does not encode a pilin nor is it required for the expression of pili, its regulation by the virulence regulator Rns implies that *cexE* encodes a virulence factor.

As in ETEC, the expression of CexE homologs in *Citrobacter rodentium* and enteroaggregative *E. coli* (EAEC) is positively regulated by virulence regulators with homology to Rns (Sheikh et al., 2002; Hart et al., 2008). In *C. rodentium cexE*_Cr_ is under the control of RegA while AggR regulates the expression of dispersin, the homolog of CexE in EAEC (Sheikh et al., 2002; Nishi et al., 2003; Hart et al., 2008). Of the three only dispersin has been characterized beyond the level of expression. Dispersin is a secreted protein that associates with the external face of the outer membrane and its NMR structure suggests that it associates with the outer membrane via electrostatic interactions (Velarde et al., 2007). Dispersin has further been shown to decrease adherence to biotic and abiotic surfaces, and reduce EAEC aggregation (Sheikh et al., 2002). Collectively these effects result in greater dispersal of EAEC *in vitro*. Whether or not these effects contribute to pathogenesis is unknown because to date there have been no *in vivo* studies of these putative virulence factors. This is partly due to the fact that ETEC and EAEC do not naturally infect small laboratory animals. However, *C. rodentium* is natural murine pathogen and a well-developed model for enteric infections (Mundy et al., 2005). Therefore, we used murine *C. rodentium* challenge studies to interrogate the function of CexE *in vivo*.

## Materials and Methods

### Bacterial Growth Conditions

Bacteria were cultured aerobically at 37°C in CFA medium (Evans et al., 1977), Lysogeny Broth (LB; HIMEDIA), or Iscove’s Modified Dulbecco’s Medium (IMDM; Thermo Fisher Scientific). For hypoxic growth bacteria were cultured in sealed CyroELITE^TM^ cyrogenic vials (Wheaton) at 37°C in IMDM. Growth media were supplemented with 50 μg/ml kanamycin, 50 μg/ml hygromycin, or 100 μg/ml ampicillin as appropriate.

### Bacterial Strains and Plasmids

Strains, plasmids, and primer sequences are listed in Tables 1–3 respectively. Plasmid pGPM1034bla expresses a CexE-TEM-1 fusion protein from *cexEp* or T7p. It was constructed by amplification of the β-lactamase gene from pUC19 with oligonucleotide primer pair 1126/1127. The PCR product was digested with XhoI then ligated into the same site of pGPM1034. Plasmid pGPM1039-27D expresses CexE from *cexEp*. It was constructed by amplification of the cexE gene from 394/478. The PCR product was digested with BamHI and XbaI then ligated into the previously digested pNEB193 vector. HiFi DNA assembly was used to construct plasmid pTags2-cexC-1D4 and pTags2-cexC_Cr_-FLAG which expresses CexC-1D4 (∼24 kDa) and CexC_Cr_-FLAG (∼27 kDa), respectively, from *lacp*. For cexC the vector backbone was amplified from pTags2 with primers 1533/1534. *CexC* was amplified from H10407 with primers 1531/1532. For *cexC*_Cr_ the vector backbone was amplified from pTags2 with primers 1415/1416. *CexC*_Cr_ was amplified from DBS100 with primers 2142/2143. The PCR products were assembled with NEB HiFi. λRED mediated recombineering was used for the construction of the following strains as previously described (Datsenko and Wanner, 2000; Datta et al., 2006). Kanamycin resistance cassettes targeting *cfaD* and *cexE*_α_ were amplified from pKD4 with primer pairs 608/625 and 622/623, respectively. A cassette for the disruption of the *lac* operon was amplified from JF876 with primers 108/518. Electroporation of the cassettes into H10407 / pSIM6 resulted in recombinants GPM1236, GPM1163, and GPM1168, respectively. Cassettes for epitope tagging, disruption, or insertion downstream of *cexE*_Cr_ were amplified from pSUB11 with primer pairs 1178/1179, 1177/1179, and 1193/1179, respectively. Electroporation of the cassettes into DBS100/pSIM6 resulted in recombinants GPM1830, GPM1827, and GPM1831a, respectively. Cassettes for disrupting *cexC*_Cr_ were amplified from pSUB11 with primer pair 1673/74 and pTags2 with primer pair 2136/37. Electroporation into DBS100 / pSIM6 resulted in recombinant GPM2002 and GPM3152, respectively. Recombinants were cured of λRED expression plasmids by passage at 42°C. Insertions were verified by PCR with primers flanking the insertion sites.

**TABLE 1:** Strains used in this study.

Strain	Characteristics	Source
H10407	ETEC O78:H11	Crossman et al., 2010
JF876	ETEC Δ lacZYA514::kan	Dorsey et al., 2006
GPM1163	H10407 *cexE_α_::kan*	This study
GPM1168	H10407 Δ *lacZYA514::kan*	This study
GPM1236	H10407 *rns::kan*	This study
GPM1820a	H10407 *cexC::kan*	This study
DBS100	*Citrobacter rodentium* (ATCC 51459)	Schauer et al., 1995
GPM1830	DBS100 *cexE_Cr_-FLAG kan*	This study
GPM1831a	DBS100 *cexE*_Cr_ Ω (66bp::*kan*), intergenic kan insertion downstream of *cexE*_Cr_	This study
GPM1827a	DBS100 *cexE_Cr_::kan*	This study
GPM1827b	DBS100 *cexE_Cr_::kan*	This study
GPM2002a	DBS100 *cexC_Cr_::kan*	This study
GPM2002b	DBS100 *cexC_Cr_::kan*	This study
GPM3152	DBS100 *cexE_Cr_-FLAG::kan cexC_Cr_::hyg*	This study

**TABLE 2:** Plasmids used in this study.

Name	Description	Marker	Source
pNEB193	Cloning vector	Amp	New England Biolabs
pUC19	Cloning vector	Amp	Yanisch-Perron et al., 1985
pGPM1034	CexE-His6 expressed from T7p or *cexEp*	Kan	Pilonieta et al., 2007
pGPM1034bla	CexE-TEM-1-His6 expressed from T7p or *cexEp*	Kan, Amp	This study
pGPM1039-27D	CexE expressed from *cexEp*	Amp	This study
pKD4	PCR template for *kan* cassettes	Amp, Kan	Datsenko and Wanner, 2000
pSIM6	λ RED expression plasmid	Amp	Datta et al., 2006
pSUB11	PCR template for 3XFLAG epitope tagging by λ RED	Kan	Uzzau et al., 2001
pTags2	Cloning vector	Amp	Addgene
pTags2-cexC-1D4	CexC_Ec_-1D4 expressed from *lacp*	Amp	This study
pTags2-cexC_Cr_-FLAG	CexC_Cr_-FLAG expressed from *lacp*	Amp	This study

**TABLE 3:** Oligonucleotide primers used in this study.

Name	Sequence
108	AGCGGAACGGGAAGGCGA
394	GCTGGATCCCGAGCGGCGTATAAAA
478	CGCTCTAGAAACATTTTACATAATGTAATCA
518	GGCTGATGATCATAACCCTGCGTTTTGCA
608	**GATTCATAAATACACTGTATTATATACATCAAATTGTATT**GTGTAGGCTGGAGCTGCTTC
622	**CAATAACCGCCCGAGCAAACAGCAAGGGCGATTTTGTTATCTT**GTGTAGGCTGGAGCTGCTTCG
623	**GGTAATTCTGAACGACCGCCTTCCGTTGCAGCAGGGGAGTG**CCATATGAATATCCTCCTTA
625	**TCAAGTAGTCAAACACTCTAGATAACAACAGTATTGGCGC**CCATATGAATATCCTCCTTA
1126	**GCAGATCTCGA***G*CACCCAGAAACGCTGGTGAAAG
1127	**CTAGTACTCGAG**TTACCAATGCTTAATCAGTGAG
1177	**GAGGTTGCATATATGAAACTTATAGGAAAATTCATTGGTTTTGCGATTATGACAATTAGC**GACTACAAAGACCATGACGG
1178	**GGCTCAGTTAACGGATATCCAGCAAAGCTTGCCACTATGCCAATTTTTAGGTGGGAATCA**GACTACAAAGACCATGACGG
1179	**CATATTGGCGCAACAATATTAACTCATAAACCTACGCATAATGTGAAGTAGAAAATATTT**CATATGAATATCCTCCTTAG
1193	**GCGAAAGCATATTGCTGTACAAAAATCAAAGTGCTCGTCATATGTTAACATGACGAGCAC**GACTACAAAGACCATGACGG
1415	**GACTACAAAGACCATGACGGTG**
1416	**CATATGTATATCTCCTTCTTGCTAGC**
1531	**GCCGCAAGGACCATAGATT**ATGATTAAATTAAGTATTGATGAGAAAATA
1532	**GACCTGGGACGTCTCCGTGCT**AAAGGATATAACCTTCATATCAC
1533	CAT**AATCTATGGTCCTTGCGGC**C
1534	**AGCACGGAGACGTCCC**
1673	**ATGATTAATTTATATATTCATAAAAAGAAATTCAGAGATAAAACCATTCTTATTGATACT**GACTACAAAGACCATGACGG
1674	**AGCTTAGCCTACTACCTCCTCATAACAAATTACAAGAAATTATCAAATGGAAGGTAAGAA**CATATGAATATCCTCCTTAG
2142	**AGCAAGAAGGAGATATACATATG**GAGATAGCGCTTCCTTACAG
2143	**CCGTCATGGTCTTTGTAGTC**ACAATTACTAAGCGTTATAACTTC

*Bold denotes sequences homologous to genomic targets for λ RED mediated or targets for HiFi assembly.

### Quantitative Bacterial Adherence Assays

HCT-8 cells, a human ileocecal epithelial cell line, was purchased from the American Type Culture Collection (ATCC CCL-244). HCT-8 cells were grown in IMDM supplemented with 10% fetal bovine serum (FBS) at 37°C in 5% CO_2_. Approximately 24 h before infection HCT-8 cells were seeded in 24-well tissue culture plates at ca. 10^5^ cells per well. Bacteria were cultured in CFA medium to an OD_600_ of 0.7–0.8. Bacteria were collected from 500 μl of culture by centrifugation then resuspended in 1 ml IMDM, 10% FBS. The tissue culture wells were aspirated, washed once with PBS, then 200 μl IMDM, 10% FBS was added to each well followed by 50 μl of the bacterial suspension for an MOI of ca. 50. After a 1 h attachment period culture medium was aspirated and wells were washed four times with PBS to remove nonadherent bacteria. Adherent bacteria were collected in PBS, 0.1% (vol/vol) Triton X-100 and enumerated by serial dilution and plating. To control for differences between inocula adherent CFUs were normalized to inocula.

### Trichloroacetic Acid (TCA) Precipitation of Culture Supernatants

H10407/pNEB193 was cultured in either CFA or IMDM to stationary phase. Bacteria were removed from the culture medium by centrifugation and successive filtration through 0.45 and 0.20 micron syringe tip filters. TCA was added to the clarified supernatants at a final concentration of 10% (vol/vol). Proteins were precipitated overnight at 4°C then pelleted for 8 min. at 21,000 × *g*. Protein pellets were washed twice with 500 μl ice-cold acetone to remove residual TCA, air-dried for 15 min. at room temperature, then resuspended in 60 μl of 1x SDS-PAGE loading buffer with 100 mM β-mercaptoethanol.

### Proteinase K Digests

Bacteria were collected from 400 μl of stationary phase cultures by centrifugation at 21,000 g for 3 min. and resuspended in 75 μl of either PBS or PBS, 10 mM EDTA, 1% (vol/vol) Triton X-100. Suspensions were equilibrated at 37°C for 1 h prior to addition of proteinase K to a final concentration of 200 μg/ml. Some samples received 11 mM phenylmethylsulfonyl fluoride (PMSF) prior to enzyme addition. All samples were incubated for 1 h at 37°C with continuous gentle rocking. Enzyme activity was terminated by addition of PMSF followed by 25 μl of 4x SDS-PAGE loading buffer with β-mercaptoethanol.

### Immunoblots

Whole-cell lysates were subject to SDS-PAGE, transferred to PVDF membranes, and blocked in TBS-Blotto (25 mM TrisCl pH 7.6, 150 mM NaCl, 5% (wt/vol) powdered nonfat milk. Antibodies against CexE_α_ were produced by immunization of rabbits (Proteintech Group, Inc.) with purified CexE_α_ -His6 and used at a dilution of 1:5000 in TBS-Blotto with 0.05% vol./vol. Tween20 (Pilonieta et al., 2007). Anti-DnaK (ab69617) and anti-β-lactamase (ab12251) antibodies were purchased from AbCam and used at dilutions of 1:10,000. Anti-FLAG (F1804) was purchased from Sigma-Aldrich and used at a dilution of 1:10,000 HRP conjugated goat anti-rabbit (sc-2030) and goat anti-mouse (115-036-062) antibodies were purchased from Santa Cruz Biotechnology and Jackson ImmunoResearch Laboratories, respectively. Secondary antibodies were used at a dilutions of 1:10,000. Chemiluminescence was detected with an Odyssey FC Imaging System (LI-COR Biosciences). Densitometry analysis was performed utilizing ImageJ software.

### *In vivo* Studies

All mice were bred and housed under barrier conditions in the Division of Veterinary Resources of the University of Miami Miller School of Medicine. Mice were regularly screened for specific common pathogens. C57Bl/6 mice were inoculated orogastrically with *C. rodentium* strains in PBS. Adults were inoculated with a 22-gauge, round-tipped feeding needle. Infant mice were used at 15 days of age and inoculated with PE-10 tubing (polyethylene tubing with an outside diameter of 0.61 mm) attached to a 30-gauge needle. Administered CFUs were determined by serial dilution and plating of inocula. Infant mice were weaned at 3½ weeks of age. On select days fecal pellets were collected from adult mice and homogenized in sterile ddH2O, diluted, and plated on MacConkey agar with kanamycin. Organs of adult mice were homogenized in PBS using a OMNI International Tissue Homogenizer (Kennesaw, GA, United States) for 2 min at medium speed. Organs from infant mice were homogenized in HBSS using a Seward Biomaster 80 Stomacher (Brinkman, Westbury, NY, United States) for 4 min. at high speed. Homogenates were diluted and plated on MacConkey agar plates with kanamycin. CFUs were normalized to the weight of fecal pellets and organs.

### Histopathology Scoring

The distal 1 cm of the colons of infected mice were collected and fixed in 10% neutral buffered formalin. The fixed distal colons were embedded in paraffin, sectioned into three equal parts, and stained with hematoxylin and eosin. Total surface area of the sections were blindly scored twice using light microscopy by evaluation of submucosal edema, PMN infiltration, and epithelial necrosis. Briefly, pathology was scored as WNL (0, <1% of surface area affected), mild (1, <5% surface area affected), moderate (2, 5–10% surface area affected), severe (3, 10–40% surface area affected), and extensive (4, >40% surface area affected). Samples scored differently amongst rankings with a difference of 1 were averaged. No samples differed by more than one rank between the duplicate scorings. This method was adapted from (Yu et al., 2017).

### Growth Curves

To test if the kanamycin insertion into the *cexE* or *cexC* genes disrupts bacteria we constructed growth curves of WT bacteria and compared them to their respective mutants. Due to the variation in bacterial size biomass indicators such as optical density can be misleading (Swinnen et al., 2004). Because of this we used viable count measurements via serial dilution and plating to accurately determine population lag times. Fifty microliter baffled flasks of IMDM were inoculated with bacteria and grown over at 37°C with agitation at 200 rpm. The bacteria were then diluted to an OD_600_ of 0.05 in new baffled flasks with fresh IMDM. Approximately every 30 min samples were taken and enumerated by serial dilution and plating. Growth curves were obtained by plotting CFU/ml against time. Slopes of the bacterial growth curve were analyzed by nonlinear regression to generate a semilog line.

### Statistical Analysis

One-way ANOVA with Tukey’s multiple comparisons test, Student’s *t*-test, Mann-Whitney *U*-test, nonlinear regression, and Mantel-Cox log rank test. (GraphPad Prism Version 8.1.2 was utilized for statistical analysis).

### Ethics Statement

All animal experiments were approved by and performed in accordance with the University of Miami Institutional Animal Care and Use Committee guidelines.

## Results

### CexE Is Broadly Distributed Across ETEC Strains

CexE was initially identified as a member of the Rns regulon by an *in silico* search of the genome of ETEC strain H10407 for Rns binding sites (Pilonieta et al., 2007). In the intervening years over 200 ETEC genomes have been sequenced and deposited in NCBI’s databases; most as whole genome shotgun (WGS) sequences. To gauge the distribution of CexE across ETEC strains we ran iterative BLASTN searches to identify *cexE* homologs. After each search the most distal member was used as the query for a subsequent search until no additional members were found. BLASTP searches were not effective because automated annotation pipelines apparently have a high failure rate with regards to the identification of *cexE* alleles. The reasons for this are unclear as these genes are open reading frames of ca. 360 bp; most with recognizable Shine-Dalgarno sequences upstream of ATG start codons.

**FIGURE 1:**
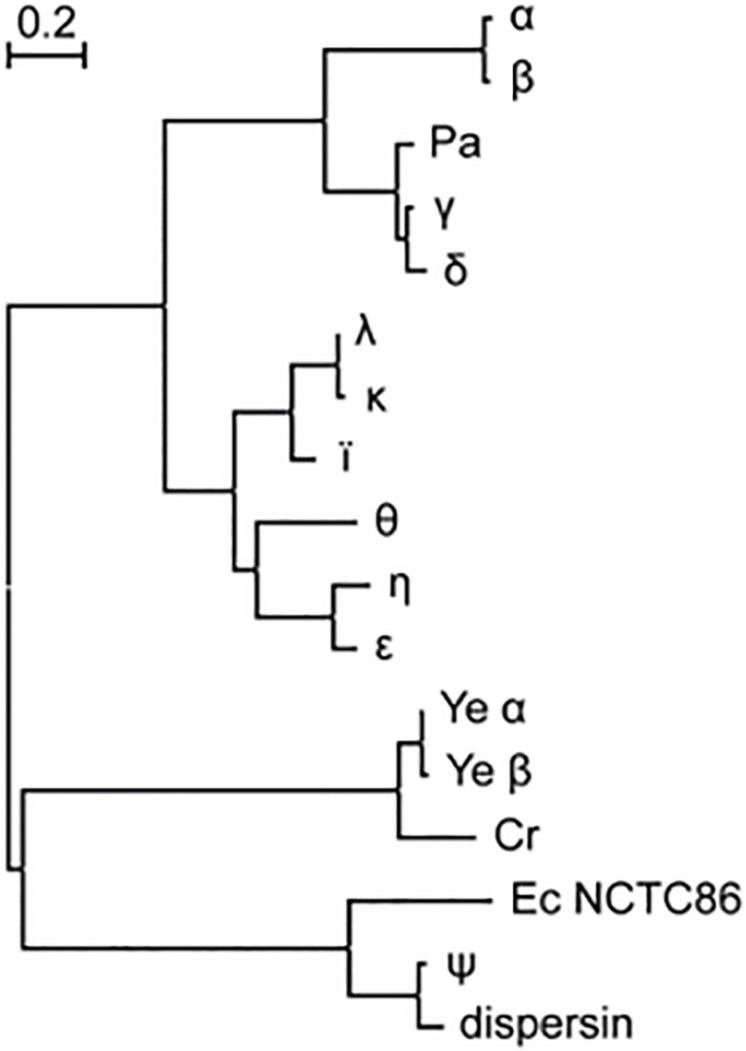
CexE homologs are prevalent amongst ETEC and a subset of other enteric pathogens. Proteins from ETEC strains are designated with a single Greek letter. dispersin is from EAEC strain 042. Ec NCTC86 is the strain of *E. coli* that was originally isolated by Theodor Escherich in 1885. The phylogram was constructed using the maximum likelihood method on a multiple sequence alignment generated by the MUSCLE algorithm; both of which were run within the MEGA7 software package (22). Cr, *C. rodentium*; Pa, *P. alcalifaciens;* Ye, *Y. enterocolitica.*

Of the 250 strains that were identified as ETEC –either explicitly in the record definition lines or by the presence of genes encoding the heat-labile (LT) and/or heat-stable (ST) enterotoxins– 76% were found to harbor *cexE* alleles. The prevalence of *cexE* alleles is similar to the genes encoding LT and ST which are present in 64 and 78% of ETEC strains, respectively. The 190 alleles of *cexE* encode eleven distinct polypeptides (Figure 1). Hereafter these will be distinguished from each other by Greek letter subscripts starting with CexE_α_ that was first identified by Pilonieta et al. (2007). Protein sequences and multi-sequence alignment are presented in Supplementary Figures S1, S2. Each polypeptide is likely transported to the periplasm via the general secretory pathway because each contains a predicted signal peptide at its amino terminus (Pugsley, 1993; Bendtsen et al., 2004). For CexE_α_, this prediction has been confirmed experimentally (Pilonieta et al., 2007).

With the lone exception of CexE_ψ_ all ETEC variants of CexE cluster within the same clade. Within the CexE_ψ_ clade are two homologs that were found in 60% of the 162 *Yersinia enterocolitica* strains in NCBI’s WGS databases. In contrast to the situation in ETEC there is little variation between *Y. enterocolitica* strains; 93% of the polypeptides are identical to Ye_α_ found in a strain of *Y. enterocolitica* (ERL04757) that was isolated from human blood (Reuter et al., 2014). Other species of *Yersinia* apparently lack CexE homologs. A CexE homolog is also present in the two complete genomes of *Citrobacter rodentium* as well as in one isolate of *Providencia alcalifaciens*. The former is a murine enteric pathogen while the latter has been described as an emerging enteric pathogen of humans (Schauer and Falkow, 1993; Murata et al., 2001; Yoh et al., 2005). Notably the *P. alcalifaciens* homolog resides within a clade of ETEC proteins while the *Y. enterocolitica* and *C. rodentium* homologs are more closely related to dispersin of enteroaggregative *E. coli* (EAEC) (Figure 1). Also within the dispersin clade is CexE_ψ_ from ETEC as well as a polypeptide from *E. coli* strain NCTC86. The latter was isolated by Theodor Escherich in 1885 as he sought to identify the cause of neonatal dysentery (Méric et al., 2016; Khetrapal et al., 2017).

### CexE_α_ Is a Conditional Coat Protein in ETEC

**FIGURE 2:**
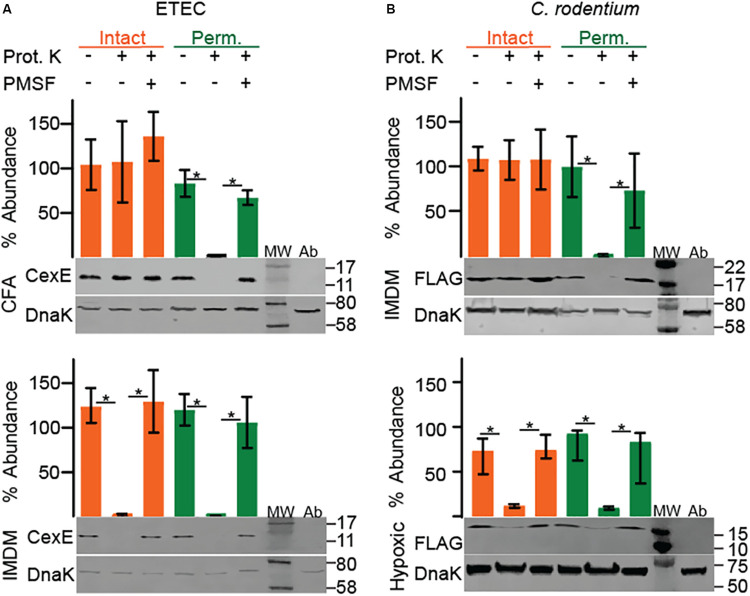
Culture medium and oxygen levels influence the subcellular distribution of CexE. Intact cells that present CexE on their outer membranes render it sensitive to proteinase K digestion as determined by Western blots and quantification. Cells that require permeabilization for digestion of CexE have failed to transport it across the outer membrane. **(A)** Permeabilization was required for the digestion of CexE_α_ when ETEC H10407 was grown in CFA but not IMDM. **(B)** FLAG tagged CexE_Cr_ was retained within the envelope of *C. rodentium* when GPM1830 was cultured aerobically in IMDM but not when cultured in IMDM under hypoxic conditions. Representative blots are shown. Quantification of CexE digestion relative to DnaK. For quantification *n* = 3, *P < 0.05 by Student’s *t*-test. Prot. K, proteinase K; MW, molecular weight ladder; Ab, antibody specificity controls. Antibody specificity control lanes were loaded with whole cell lysates of ETEC strain GPM1163 (*cexE*_α_*::kan*) or *C. rodentium* strain DBS100 which lacks the FLAG epitope.

**FIGURE 3:**
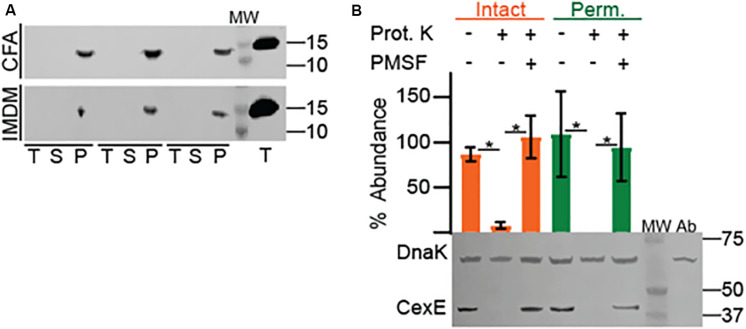
Association of native CexE_α_ and a 43 kDa CexE_α_ -TEM-1 fusion protein with the outer membrane of ETEC. **(A)** H10407 was cultured in either CFA or IMDM. Cell pellets (P), culture supernatants (S), and TCA precipitates (T) probed for CexE_α_. The far-right lane contains the TCA precipitate of 10ng of purified CexE_α_ -H6. **(B)** Translocation of CexE_α_ -TEM-1 (β-lactamase) to the surface of ETEC cultured in IMDM. Representative Western blot of whole cell lysates of ETEC strain GPM1163 (*cexE_α_ ::kan*)/pGPM1034bla probed with anti-CexE and DnaK antibodies. Quantification of CexE_α_ -TEM-1 digestion relative to DnaK. For quantification *n* = 3, *P < 0.0001 by Student’s *t*-test. Prot. K, proteinase K; MW, molecular weight ladder; Ab, antibody specificity control with whole cell lysates of GPM1163 lacking pGPM1034bla.

Like CexE_α_, dispersin of EAEC has an amino terminal signal peptide and enters the periplasm through the general secretory pathway (Pilonieta et al., 2007). Subsequently dispersin crosses the outer membrane but remains associated with the cell as a coat protein bound by electrostatic interactions (Sheikh et al., 2002; Velarde et al., 2007). To determine if CexE_α_ and CexE_Cr_ are also coat proteins proteinase K was used as a membrane impermeable probe of their subcellular distribution. For these experiments we also chose culture media that resulted in moderate to robust expression of the target proteins. When ETEC was cultured in IMDM CexE_α_ associated with intact and permeabilized cells was digested by the protease with equivalent efficiency (Figure 2A, bottom). This indicates that the majority of CexE_α_ is deposited on the outer membrane and that there is little to no intracellular pool. In addition the amount of cell-free protein was negligible as it was below detection even after TCA precipitation of IMDM supernatants (Figure 3A). However, the translocation of CexE_α_ across the outer membrane is conditional because it remained intracellular when ETEC was cultured in CFA medium (Figure 2A, top).

In contrast, CexE_Cr_-FLAG was not translocated across the outer membrane when *C. rodentium* was cultured in IMDM or LB (Figure 2B, top and Supplementary Figure S3). It is unlikely that the <3 kDa FLAG epitope interfered with the translocation of CexE_Cr_ because fusion of TEM-1 –a 30 kDa protein– to the carboxy terminus of CexE_α_ did not inhibit its translocation (Figure 3B). We also did not observe secretion of CexE_Cr_-FLAG when *C. rodentium* was cultured in CFA with 45 mM NaHCO_3_, or LB with 45 mM NaHCO_3_ in the presence or absence of 4 mM bile salts. Culturing on LB agar plates also failed to induce secretion. Likewise CexE_Cr_-FLAG was not secreted in M9 minimal media supplemented with 0.4% glucose or glycerol, DMEM, or RPMI with GlutaMAX^TM^. Varying the temperature from 30° to 42°C also had no effect. We also unsuccessfully attempted to induce CexE_Cr_ secretion using unconventional methods such as culturing *C. rodentium* in homogenates of C57Bl/6 intestinal tissue and/or feces to mimic *in vivo* conditions. However when *C. rodentium* was cultured under hypoxic conditions CexE_Cr_ associated with intact cells was efficiently digested (Figure 2B bottom). Thus, reduced oxygen levels –as would be expected in lumen of the large intestine– induces the secretion of CexE_Cr_ to the outer membrane. These results also demonstrate that the secretion of CexE is not linked to its expression. Rather secretion is regulated and responsive to environmental cues and/or growth conditions. Although secretion conditions differ between ETEC and *C. rodentium*, secreted CexE remains predominantly associated with the outer membranes of both species.

### CexE_α_ and CexE_Cr_ Do Not Significantly Modulate Adherence

**FIGURE 4:**
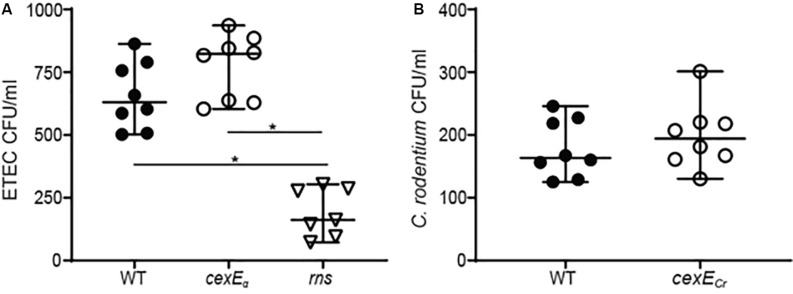
CexE_α_ and CexE_Cr_ do not significantly modulate adherence to mammalian cells. Adherent bacteria were enumerated after a 1 h infection of HCT-8 cells in IMDM 10% fetal bovine serum. **(A)** The difference between GPM1168 (WT ETEC) and a GPM1163 (*cexE*_α_ ::*kan*) was not statistically significant. In contrast, GPM1236 (*rns::kan*) was significantly impaired in its ability to adhere to HCT-8 cells. **(B)** The difference between GPM1831a (WT *C. rodentium*) and GPM1827a (*cexE*_Cr_*::kan*) was not statistically significant. Graphs display median and 95% CI. Symbols represent samples collected from separate wells. *P < 0.0001 by Tukey’s multiple comparisons test.

Since CexE_α_ is a coat protein we sought to determine if it affects adherence. Prior to doing so we determined that the growth rates of ETEC and *C. rodentium cexE* mutants were equivalent to the WT parent strain of each (Supplementary Figures S4A–C). In adherence assays we observed that a *cexE*_α_ mutant was more adherent to HCT-8 cells than WT (Figure 4A). However, the difference was insignificant and in stark contrast to the effects of an insertion in *rns* that abolishes expression of both CexE_α_ and the adhesive CFA/I pilus. These results suggest that CexE_α_ does not significantly modulate the adherence of ETEC to HCT-8 cells even when the pathogen is cultured in IMDM; a condition in which CexE_α_ coats the outer membrane. The difference between WT *C. rodentium* and a *cexE*_Cr_ mutant was also insignificant (Figure 4B).

### CexC Is Necessary for the Secretion of CexE

**FIGURE 5:**
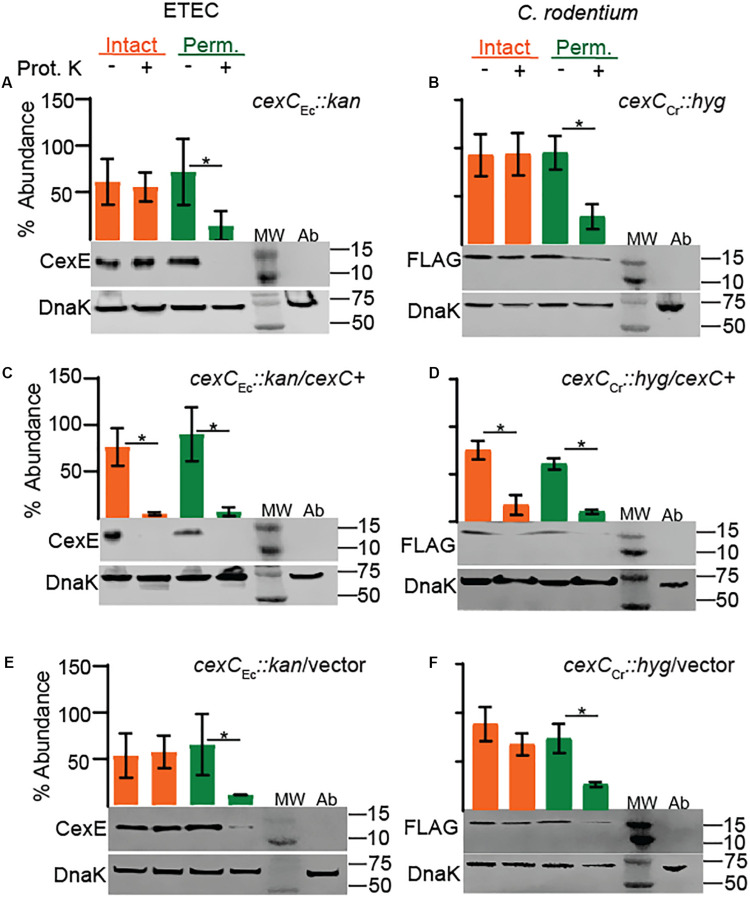
CexC is required for the secretion of CexE in both ETEC and *C. rodentium*. CexE secretion is abolished in *cexC* mutants of **(A)** ETEC strain GPM1820a and **(B)** *C. rodentium* strain GPM3152. Complementation with CexC expression plasmids **(C)** pCexC-1D4 or **(D)** pCexC-FLAG restored secretion of CexE to the outer membrane of both pathogens. CexC_Cr_-FLAG is not visible because it is approximately 27 kDa. **(E,F)** As expected the vector control pTags2 failed to restore secretion. Quantification of CexE_α_ digestion relative to DnaK. For quantification *n* = 3–4, *P < 0.05 by Student’s *t*-test. Prot. K, proteinase K; MW, molecular weight ladder; Ab, antibody specific controls. Antibody specificity control lanes were loaded with whole cells lysates of ETEC strain GPM1163 (*cexE*_α_ *::kan*) or *C. rodentium* strain DBS100 which lacks the FLAG epitope.

In EAEC the secretion of dispersin is dependent on the Aat secretion system encoded by *aatPABCD* (Nishi et al., 2003) and homologs of each gene are present in both ETEC and *C. rodentium*. Although *aatPABCD* are contiguous in the prototypical EAEC strain 042 (Nishi et al., 2003), in both ETEC and *C. rodentium cexPABC* and *cexD* are always separated by at least several KBs and in some strains they occur on separate virulence plasmids. To test if CexE secretion is dependent on the putative Cex secretion system we chose to disrupt *cexC* since insertions in the terminal gene of the *cexPABC* operon would not have polar effects. As expected secretion of CexE was abolished in *cexC* mutants of both ETEC and *C. rodentium* because CexE was not digested by proteinase K unless their outer membranes were chemically permeabilized (Figures 5A,B). Complementation of the *cexC* mutants restored translocation of CexE across the outer membranes of both pathogens but vector controls did not (Figures 5C–F). Thus, CexC –the putative ATPase of the Cex secretion system– is required for the secretion of CexE in both ETEC and *C. rodetium*; as is the case for AatC and dispersin of EAEC (Nishi et al., 2003).

### CexE_Cr_ Is a Secreted Virulence Factor *in vivo*

**FIGURE 6:**
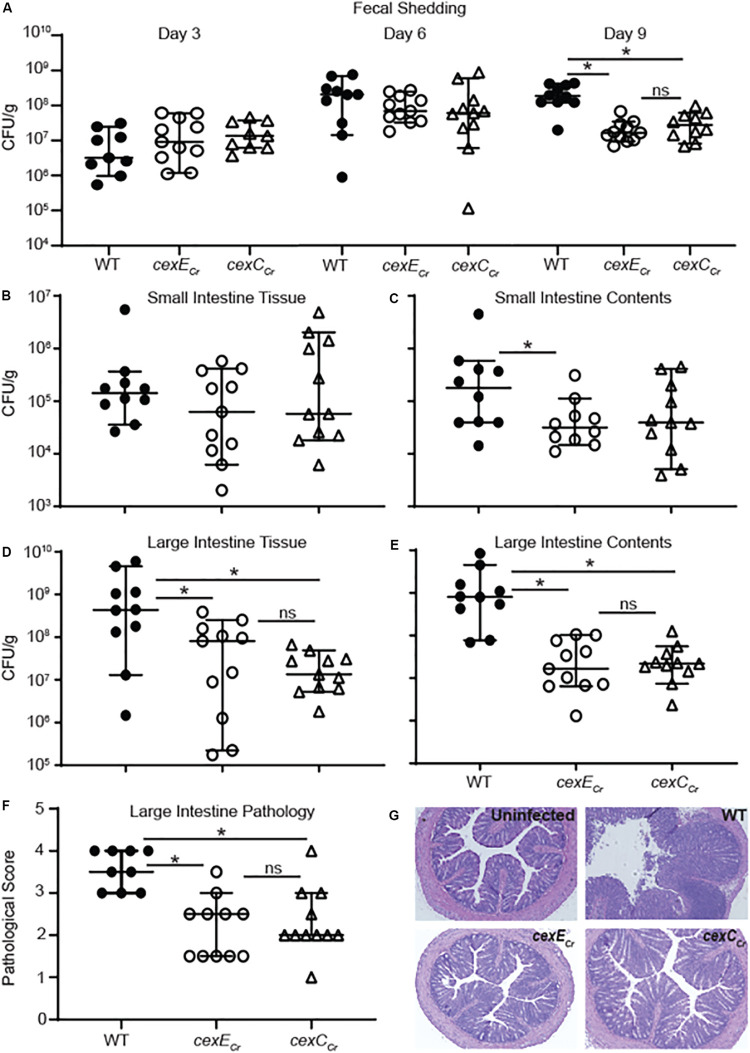
Intestinal and fecal loads of mice infected with WT *C. rodentium* and *cex* mutants. C57BL/6 mice were orogastrically inoculated with 10^8^ CFU of either GPM1831a (WT *C. rodentium*), GPM1827a (*cexE_Cr_::kan*), or GPM2002a (*cexC_Cr_::kan*). **(A)** Fecal pellets were collected on the days indicated post-inoculation. **(B–E)** Intestinal tissue and contents were harvested 10 days post-inoculation. CFUs were normalized to sample mass. **(F,G)** Histological scoring and representative images of H&E-stained colon sections at day 10. Medians and 95% CI are shown. *n* = 9–10 mice per group, *P < 0.05 by Mann-Whitney *U*-test.

To determine whether or not CexE is a virulence factor we used *C. rodentium* for *in vivo* studies because it is a natural murine pathogen; unlike EAEC and ETEC. We orogastrically inoculated C57BL/6 mice with *C. rodentium* strains GPM1831a –which carries a kanamycin cassette in an intergenic region downstream of *cexE* and was considered our WT strain– or GPM1827a (*cexE_Cr_::kan*). We inoculated a third group of mice with GPM2002a (*cexC_Cr_::kan*) to evaluate if CexE_Cr_ is secreted *in vivo*. As gauged by fecal shedding on days three and six post-inoculation, the three strains of bacteria colonized the mice equally well (Figure 6A). However the amount of shed WT bacteria continued to increase while that of the mutants plateaued, resulting in significant differences between WT and mutant strains on day nine (Figure 6A). Not surprisingly, day nine fecal shedding was mirrored by the intestinal loads of WT and mutant strains on day ten; particularly in the large intestine where *C. rodentium* preferentially colonizes (Figures 6B–E; Silberger et al., 2017). The results were reproduced in another infection experiment, with an independent *cexE*_Cr_ mutant (GPM1827b) (Supplementary Figures S5A–C). We also observed and scored the pathology of the distal large intestine and found that that WT bacteria were associated with more intestinal pathology than either mutant (Figures 6F,G and Supplementary Figure S5D).

Across all measurements the differences between the *cexE*_Cr_ and *cexC*_Cr_ mutants were statistically insignificant (Figure 6). This suggest that CexC_Cr_ is required for secretion of CexE_Cr_ *in vivo* and is consistent with our *in vitro* results (Figure 5). A separate experiment with an independent *cexC*_Cr_ mutant (GPM2002b) replicated the day 9 fecal shedding results accompanied by a trend of fewer mutant bacteria in the large intestine and less pathology than the WT strain (Supplementary Figure S6). Over the course of these infections mice were also monitored for weight loss. Consistent with previous *C. rodentium* murine studies, we observed only mild and transient weight loss (Bhinder et al., 2013). We further disaggregated the data based on the sex of the animals and found no trends to suggest a gender-bias in infections (data not shown). Additionally these results were not restricted to C57BL/6 mice as similar results were observed when 129X1/SvJ mice were used (Supplementary Figure S7).

**FIGURE 7:**
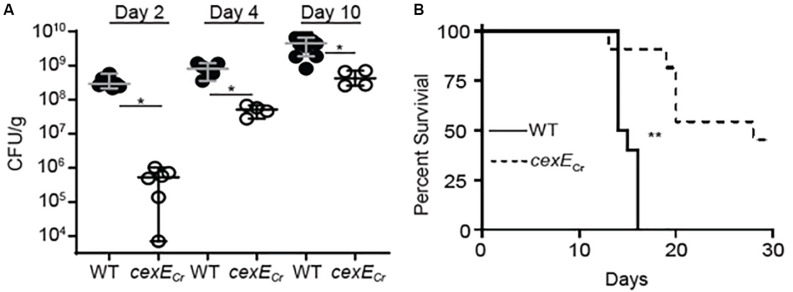
*C. rodentium cexE*_Cr_ mutants are attenuated in neonatal mice. Fifteen-day old C57Bl/6 mice were infected with 10^7^ CFU of GPM1831a (WT *C. rodentium*) or a GPM1827a (*cexE_Cr_::kan*) in groups of 12 and 6, respectively. **(A)** *C. rodentium* colon titers, normalized to tissue mass, were determined on the days indicated post-inoculation. Median and 95% CI are shown. *n* = 4–12 mice per group, *P < 0.05 by Mann-Whitney *U*-test. **(B)** In a separate experiment neonate survival was monitored out to 30 days post-inoculation. *n* = 10–11 animals per group, **P = 0.0005 by log rank (Mantel-Cox) test.

Our results demonstrating that CexE_Cr_ contributes the pathogenicity of *C. rodentium* were further confirmed in an infant mouse model (Figure 7). For technical reasons the small intestines were not analyzed but the loads of both the WT and mutant strain in the large intestine increased over the course of the experiment (Figure 7A). However, the loads of WT strain significantly exceeded those of the *cexE*_Cr_ mutant on each of the three sampling days (Figure 7A). Although immunocompetent adult mice are able to clear *C. rodentium* infections, all infant mice infected with the WT strain perished (Figure 7B; Diaz et al., 2017). In contrast a significant number of the mice infected with the *cexE*_Cr_ strain survived for the duration of the experiment. In aggregate these results demonstrate that CexE_Cr_ is a secreted virulence factor of *C. rodentium* that results in higher intestinal burdens and increased virulence in both adult and infant mice.

## Discussion

We have found that a majority of ETEC strains harbor *cexE* alleles. Moreover, *cexE* homologs have been acquired by another pathovar of *E. coli* –EAEC– as well as three other species of enteric pathogens; *P. alcalifaciens*, *Y. enterocolitica*, and *C. rodentium*. For consistency with prior literature *cexE* homologs are referred to as dispersin when present in EAEC (Sheikh et al., 2002). These observations suggest a selective advantage for *cexE* and dispersin’s acquisition and maintenance. Previous studies with dispersin provide much of what is known about this family of proteins but have been limited to *in vitro* studies by the lack of a suitable animal model. *In vitro* dispersin coats the outer membrane of EAEC and has been proposed to promote dispersal by counteracting autoagglutination mediated by AAF fimbriae (Velarde et al., 2007). We have also found that CexE is a coat protein in both ETEC and *C. rodentium*. However the adherence of *cexE* mutants to mammalian HCT-8 cells was not significantly different than WT ETEC. The biological relevance of this result is uncertain and requires studies with additional cell lines, or ideally with intestinal tissues. We also did not observe significant differences in agglutination (data not shown) but note that dispersin’s pronounced effects were dependent on it is artificial overexpression from the strong T5 promoter (Sheikh et al., 2002).

**FIGURE 8:**
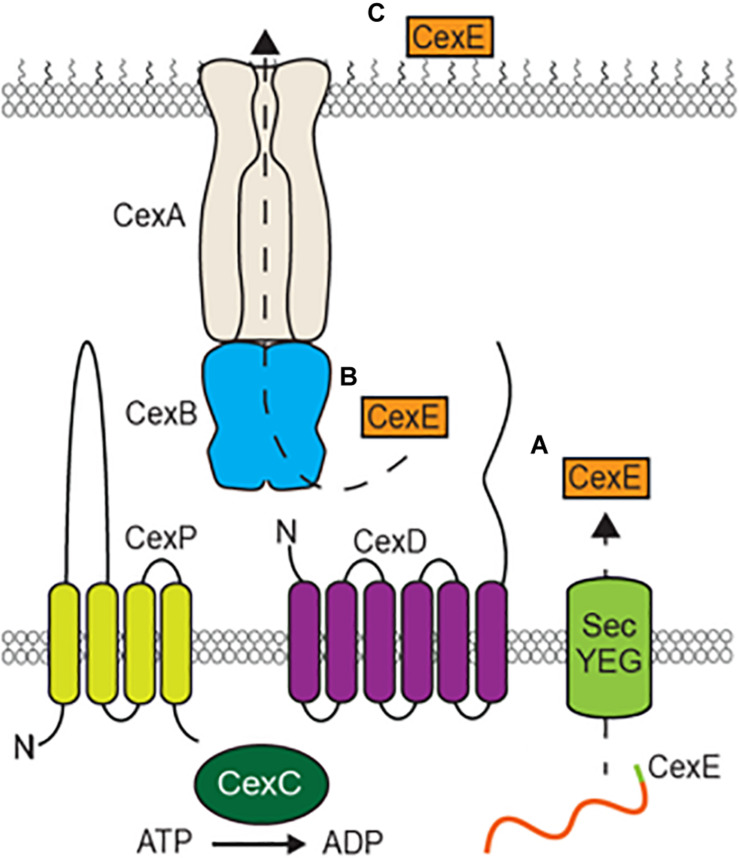
Model of CexE secretion by an atypical Type I secretion system. **(A)** The CexE polypeptide is transported from the cytoplasm to the periplasm by the SecYEG complex. **(B)** After cleavage of its signal peptide the CexPABCD secretion complex then transports CexE across the outer membrane **(C)** which it subsequently binds to.

All CexE variants and dispersin have predicted amino terminal signal peptides that would result in their transport to the periplasm via the general secretory pathway (Bendtsen et al., 2004). Such signal peptides are cleaved upon entry into the periplasm and cleavage of CexE_α_’s signal peptide has been experimentally verified (Pilonieta et al., 2007). This is apparently followed by a second translocation event that transports CexE to the external face of the outer membrane that is dependent upon by environmental cues and or growth conditions. For dispersin of EAEC this second translocation event requires five genes, *aatPABCD* (Nishi et al., 2003). Homologs of all five genes are also present in ETEC strains harboring *cexE* as well as *C. rodentium*, *Y. enterocolitica*, and *P*. *alcalifaciens* (unpublished observation). CexA/AatA likely form a conduit through the outer membrane because they are homologs of TolC; a protein that has been shown to form β-barrel pores in the outer membrane of *E. coli* and other gram-negative bacteria (Figure 8) (Koronakis et al., 2000, 2004). Type I secretion systems also utilize TolC or its homologs to transport client proteins (Wandersman and Delepelaire, 1990). However, such systems accept their clients from the cytoplasm and transport them in a single translocation event (Green and Mecsas, 2015). In contrast the secretion of both CexE and dispersin apparently involves two steps; one across the inner membrane through the SecYEG complex and a second across the outer membrane via their cognate secretion systems. CexB/AatB are predicted to be periplasmic proteins with amino-terminal signal peptides; although, some models suggest that their amino-termini are instead transmembrane helices (Tsirigos et al., 2015). CexP/AatP and CexD/AatD are predicted to be inner membrane proteins with significant extensions in the periplasm (Tsirigos et al., 2015). CexC/AatC are predicted to be cytoplasmic proteins and may energize translocation across the outer membrane because they contain the signatures of ATP binding cassettes (Nishi et al., 2003).

As was previously observed with dispersin (Nishi et al., 2003), we found that translocation of CexE across the outer membrane was abolished in *cexC* mutants of both ETEC and *C. rodentium*. Cex-dependent secretion also takes place *in vivo* because we observed that *cexE*_Cr_ and *cexC*_Cr_ mutants produced similar attenuated phenotypes. We also found that the secretion of CexE is not directly linked to its expression. Rather CexE accumulates in the periplasm unless the appropriate secretion conditions are met. The conditional secretion of CexE is also species specific since we observed that hypoxia triggers the secretion of CexE in *C. rodentium* but not ETEC. In either case it remains to be determined if conditional secretion is the result of differential regulation of the *cex* secretion and *cexE* genes or an actual sensing/regulatory mechanism within the secretory system.

After translocation across the outer membrane both CexE and dispersin remain associated with the bacterial envelope as coat proteins. Dispersin has been proposed to contribute to the pathogenicity of EAEC by facilitating the dispersal of the pathogen throughout the intestinal tract (Velarde et al., 2007). Although the dispersal model is an extrapolation of *in vitro* observations, it may explain the significantly higher loads of WT *C. rodentium* than the *cexE*_Cr_ and *cexC*_Cr_ mutants that we observed in the large intestines of mice. The greater fecal shedding of the WT strain compared to the mutants is likely a consequence of these higher loads. Mechanistically it has been proposed that dispersin counteracts fimbriae mediated autoagglutination (Velarde et al., 2007). However, we did not observe similar effects with our ETEC *cexE* mutants and alternative mechanisms have not yet been excluded. In particular CexE_α_ associates with ETEC outer membrane vesicles that have also been shown to facilitate the delivery of LT enterotoxin to the cytosol of mammalian cells (Horstman and Kuehn, 2000; Roy et al., 2011). Since CexE_α_ colocalizes with LT in OMVs and OMVs deliver LT to host cells it is plausible that CexE also reaches host cells. Whether or not this contributes to pathogenesis remains to be determined. Nevertheless, our *in vivo* studies clearly demonstrate that CexE promotes colonization and/or survival of an enteric pathogen within the intestinal tract.

## Data Availability Statement

The datasets generated for this study are available at NIH.FigShare.Com, https://doi.org/10.35092/yhjc.c.5015396.

## Ethics Statement

The animal study was reviewed and approved by the University of Miami Institutional Animal Care and Use Committee.

## Author Contributions

GM constructed phylogeny trees, analyzed sequence data, and obtained funding for the studies. ZR conducted *in vitro* adherence assays. ZR and KT conducted secretion assays and subsequent analyses. ZR, KT, LM, RM, and GM conducted the experiments with adult mice. BA did the experiments with neonatal mice. ZR, KT, and GM constructed the strains and plasmids. ZR, KT, LM, and GM designed the experiments and analyzed the data. ZR and GM wrote the manuscript with editorial assistance from KT, LM, and BA. All authors contributed to the article and approved the submitted version.

## Conflict of Interest

The authors declare that the research was conducted in the absence of any commercial or financial relationships that could be construed as a potential conflict of interest.
