# Supplementary material for: CexE Is a Coat Protein and Virulence Factor of Diarrheagenic Pathogens
Source: Front Microbiol. 2020 Jun 30;11:1374. doi: 10.3389/fmicb.2020.01374 (PMC7344145; doi:10.3389/fmicb.2020.01374)
Supplement: Supplementary file 1 [file Data_Sheet_1.zip › Figure S1.pdf]

Figure S1. Sequences of CexE homologs with known or putative signal peptides underlined.

>CexE$_{\alpha}$ (ETEC)
MKKYILGVILAMGSLSAIAGGGNSERPPSVAAGECVTFNSKLGEIGGYSWKYSNDACNETVAKGYAIGVAMHR
TVNYEGGYSIQSSGIVKPGSDFIMKGGKTYKGHKKVSAGGDTPYWYK
>CexE$_{\beta}$ (ETEC)
MKKYILGVILAMGSLSAIAGGGNSERQPSVAAGEGVTFNSKLGEIGGYSWKYSNDACNETVAKGYAIGVAMHR
TVNYEGGYSIQSSGIVKPGSDFIMKGGKTYKGHKKVSTGGETPYWYK
>CexE$_{\gamma}$(ETEC)
MKKYILGIILTMGSFSALAGGAWQPSVGPGECISFQSGLGEIGGYRWVSSNGCNEVINRGYALGVALERRVNY
ANGDSVSSAGVISPSRDYVMKGSKFSNGSPKVSTGGSSPYWYK
>CexE$_{\delta}$ (ETEC)
MKNYILGIILTMGSFSALAGGVWQPSVGPGECISFQPGLGEIGGYRWVSSNGCNEVINRGFALGVALERRVNY
ANGDSVSSAGVISPNSDYVIKGSKFSNGSPKVSTGGSSPYWYK
>CexE$_{\varepsilon}$ (ETEC)
MKKIILALPFLTSCFSAFAGGSGPEWQPQISPGQCIQYTEIGETGGYKWHNIDACNEVVHRGYASGAFVSGKVV
YEGGETIEYTGIVKPDAPYTIQAPSTHNGKKKVGHGGAYTYWAR
>CexE$_{\eta}$ (ETEC)
MKKIILALPFLTSCFSAFAGSSGPEWQPQISAGQCIQYTEIGEAGGYKWNNIDYCNEVVRRGYASGVFASGKVV
YEGGGVVEYTGLVKPDTPYAIQAPSTYNGKKKVGHGSAYTYWAR
>CexE$_{\theta}$ (ETEC)
MKKLFFAFPLVISSFSTFAGGGSEWQPTVSPGQCIEYTEIGETGGYKWNDTDSCNEVVRRGYASGVMVSGKV
FYDGAPSISYTGHVAPNKPYARQAPLYNDGKKKWGHGDSYTYWAR
>CexE$_{\iota}$ (ETEC)
MKKIILSLSVIVFSHSVSAGSTNWQPSVGPGQCIVYAEIGETGGYKWNNQDDCNEVVRRGYASGVGVSGRVIY
EGNTPGTNGDSIGYTGIVTPNKPYERQAPATYKGKKKVGHGDSYTYWAK
>CexE$_{\kappa}$ (ETEC)
MKRIILSLSVIFCSHSALAGSTDWQPSVGPGQRIVYAEIGETGGYKWNNQNECNEVVRRGYAIGVGVSGKVIYE
GNKPGYNGDSISYSGIVTPYRDYKRQAPAVYNGKKKVAHGDSYTYWAK
>CexE$_{\lambda}$ (ETEC)
MKRIILSLSVIFCSHSALAGSTDWQPSVGPGQCIVYAEIGETGGYKWNNQNECNEVVRRGYAIGVGVSGKVIYE
GNKPGYNGDSISYSGIVTPDRDYKRQAPAVYNGKKKVAHGDSYTYWAK
>CexE$_{\psi}$ (ETEC)
MNKIKFVIFSGILGLSLNAFAGGSGWNADNVDPSQCIKLSGVQYTYNSSASVCMQGLNEGKVRGVSVSGEFYY
NDGTTSSFKGVVTPSTPVNTNQDINKTNKVGVQKYRALTEWVK
>dispersin (EAEC)
MKKIKFVIFSGILGISLNAFAGGSGWNADNVDPSQCIKLSGVQYTYNSGVPVCMQGLNEGKVRGVSVSGVFYY
KDGTTSNFKGVVTPSTPVNTNQDINKTNKVGVQKYSALTEWVK
>Cr (*C. rodentium*)
MKLIGKFIGFAIMTISFYSFAGGGASSWIPNVAPSACVNIDESRISFTWNNNPECEKAISSGYASGVRIMGSASY
VPDTTIAQFNKVLKRNMSLTIIDLDIYGSVNGYPAKLATMPIFRWES
>Pa (*P. alcalifaciens)*
MKKYILGIILTMGSFSALAGGAWQPSVGPGECISFQSGLGEIGGYKWVSSDGCNEVINRGHALGVGLERRVNY
ANGDYVSSAGVISPNRDYVIKGSTSSNGSPKVSTGGSSPYWYK
>Ec NCTC86 (*E. coli*)
MKKLNVSIFSIAACFSLNVFAGGGGWNSDLVDPQQCVKLSGAQYTYNSSSNKCMQGINEGKVHGVSLFGTFY
YGDGSQGTFKGRVSPGTTLNTNQDMNKTNKYGVKYKVITEWVR
>Ye$_{\alpha}$ (*Y. enterocolitica*)
MKLTGKFIGFVIMTISFSSFAGGGSSSWKPSVDPSACVNINEQRTPSTWYDNPVWFTWNANPECEKAISSGYA
SGIRIMGTVSYVPESTISQFNKVLKPNMSLTIVDLDIYGAVNGHPPRFASRPEFRWES
>Ye$_{\beta}$ (*Y. enterocolitica*)
MKLTGKFIGFVIMTISFSSFAGGGSSSWKPSVDPSACVNINEQRTPSTWYDNPVWFTWNANPECEKAISSGYA
SGIRIMGTVSYVPESTISHFNKVLKPNMSLTLVDLDIYGAVNGHPPRFASRPEFRWES
