# Supplementary material for: CexE Is a Coat Protein and Virulence Factor of Diarrheagenic Pathogens
Source: Front Microbiol. 2020 Jun 30;11:1374. doi: 10.3389/fmicb.2020.01374 (PMC7344145; doi:10.3389/fmicb.2020.01374)
Supplement: Supplementary file 1 [file Data_Sheet_1.zip › Figure S3.pdf]

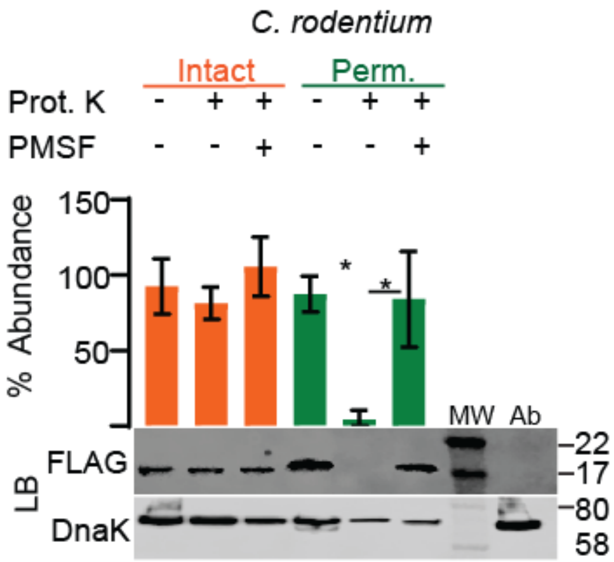

Figure S3. CexE is not secreted to the outer membrane when *Citrobacter rodentium* is cultured in LB medium.

Intact cells that present CexE on their outer membranes render it sensitive to proteinase K digestion as determined by Western blots and quantification. Cells that require permeabilization for digestion of CexE have failed to transport it across the outer membrane. Permeabilization was required for the digestion of $CexE_{Cr}$ when DBS100 was grown in LB.
