# Supplementary material for: CexE Is a Coat Protein and Virulence Factor of Diarrheagenic Pathogens
Source: Front Microbiol. 2020 Jun 30;11:1374. doi: 10.3389/fmicb.2020.01374 (PMC7344145; doi:10.3389/fmicb.2020.01374)
Supplement: Supplementary file 1 [file Data_Sheet_1.zip › Figure S4.pdf]

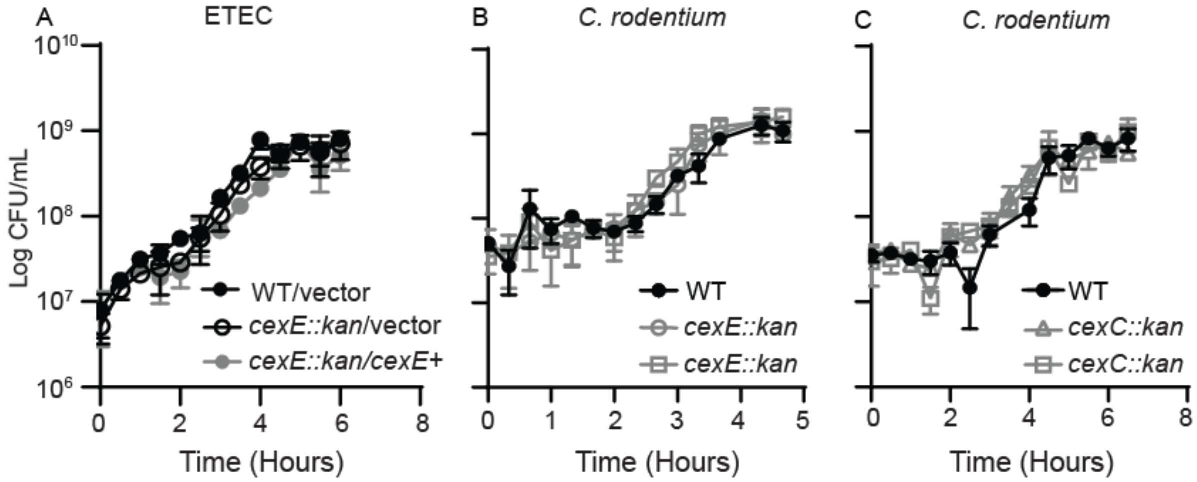

Figure S4. Figure S4. Growth curves of ETEC and *C. rodentium* strains.

Disruption of *cexE* or *cexC* genes does not affect growth rates *in vitro*. (A) Growth curves of ETEC strains H10407 (WT) and GPM1163 (*cexE::kan*) transformed with pNEB193 (vector) or pGPM1039-27D (*cexE+*). ETEC strains were cultured aerobically in IMDM at 37°C. (B) Growth curves of *C. rodentium* strains GPM1831a (WT) and $cexE_{Cr}$ mutants GPM1827a and GPM1827b. (C) Growth curves of GPM1831a and $cexC_{Cr}$ mutants GPM2002a and GPM2002b. *C. rodentium* strains were cultured aerobically in LB at 37°C. Within each experiment the differences between strains were statistically insignificant by non-linear regression.
