# Supplementary material for: CexE Is a Coat Protein and Virulence Factor of Diarrheagenic Pathogens
Source: Front Microbiol. 2020 Jun 30;11:1374. doi: 10.3389/fmicb.2020.01374 (PMC7344145; doi:10.3389/fmicb.2020.01374)
Supplement: Supplementary file 1 [file Data_Sheet_1.zip › Figure S5.pdf]

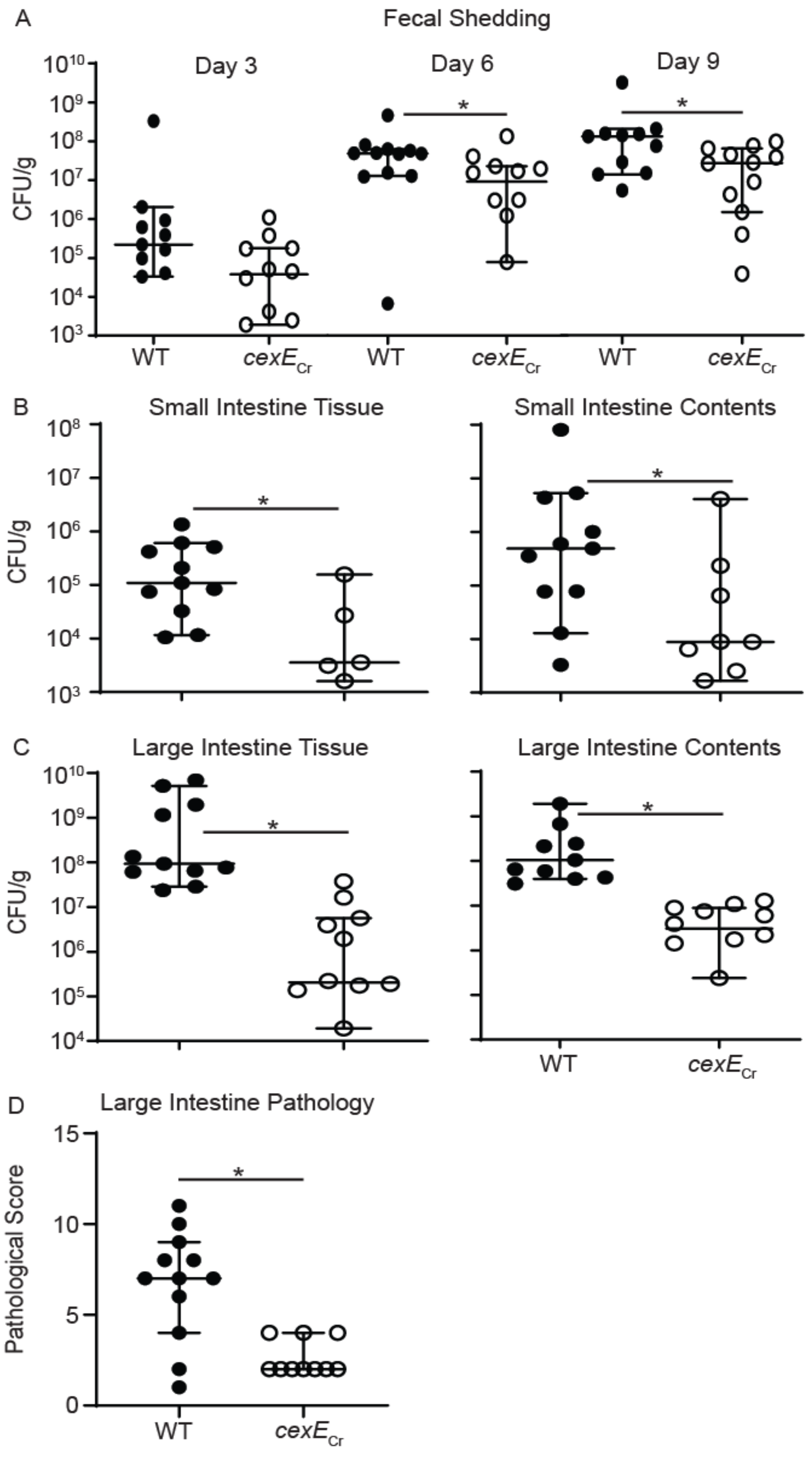

Figure S5. Fecal shedding, intestinal loads and pathology of C57BL/6 mice inoculated with wild-type *C. rodentium* and a *cexE* mutant.

C57BL/6 mice were orogastrically inoculated with 10^8 CFU of either GPM1831a (WT *C. rodentium*) or GPM1827b (*cexE*$_{Cr}$*::kan*). (A) Fecal pellets were collected on the days indicated post inoculation. (B, C) Intestinal tissue and contents were harvested 10 days post inoculation. CFUs were normalized to sample mass. (F) Histological scoring of colon sections at day 10. Medians and 95% CI are shown. n = 9-12 mice per group, *$P < 0.05$ by Mann-Whitney U test.
