# Supplementary material for: CexE Is a Coat Protein and Virulence Factor of Diarrheagenic Pathogens
Source: Front Microbiol. 2020 Jun 30;11:1374. doi: 10.3389/fmicb.2020.01374 (PMC7344145; doi:10.3389/fmicb.2020.01374)
Supplement: Supplementary file 1 [file Data_Sheet_1.zip › Figure S7.pdf]

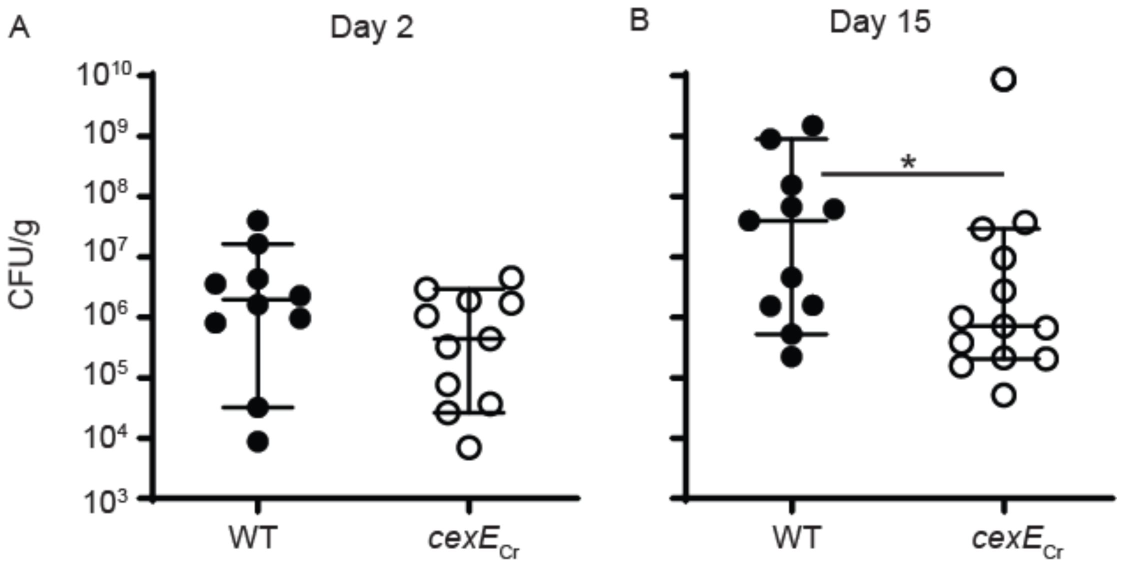

Figure S7. Fecal shedding of 129X1/SvJ mice inoculated with wild-type *C. rodentium* and a *cexE* mutant.

Fecal loads of 129X1/SvJ mice infected with WT *C. rodentium* and a *cexE*$_{Cr}$ mutant.129X1/SvJ mice were orogastrically inoculated with 10^{10} CFUs with either GPM1831a (WT *C. rodentium*) or GPM1827a (*cexE*$_{Cr}$*::kan*). Fecal pellets were collected on days 2 and 15 post inoculation and CFUs were normalized to the mass of each pellet. Although the difference between fecal shedding of the two strains was not significant at the early (A) time point it was statistically significant (B) 15 days after inoculation. Graphs display median and 95% CI. $n \geq 10$ mice per group. *$P < 0.05$ by Mann-Whitney U test
